# Scene construction ability in neurotypical and autistic adults

**DOI:** 10.1177/13623613231216052

**Published:** 2023-12-28

**Authors:** Marchella Smith, Lindsey Cameron, Heather J Ferguson

**Affiliations:** University of Kent, UK

**Keywords:** autism, scene construction, self-awareness, self-projection, social cognition

## Abstract

People with autism spectrum conditions (ASC) have difficulties mentally simulating events, perhaps due to a difficulty mentally generating and maintaining a coherent spatial scene – that is, ‘scene construction’. The current study compared scene construction ability between autistic adults (*N* = 55) and age-, gender- and Intelligence Quotient-matched neurotypical adults (*N* = 63), using a task in which participants were asked to vividly imagine and describe fictitious scenes. Results showed that scene construction was diminished in autistic compared to neurotypical participants and was negatively associated with autistic traits. ASC diagnosis did not influence the frequency of self-reference or sensory experiences, which followed the same pattern in both groups: sight was referenced more than sound, sound was referenced more than both touch and smell, which were both referenced more than taste. Exploratory analysis of some of the cognitive predictors revealed that scene construction ability was associated with individual differences in Theory of Mind and alexithymia.

**Lay Abstract:**

People with autism spectrum conditions (ASC) have difficulties imagining events, which might result from difficulty mentally generating and maintaining a coherent spatial scene. This study compared this scene construction ability between autistic (*N* = 55) and neurotypical (*N* = 63) adults. Results showed that scene construction was diminished in autistic compared to neurotypical participants, and participants with fewer autistic traits had better scene construction ability. ASC diagnosis did not influence the frequency of mentions of the self or of sensory experiences. Exploratory analysis suggests that scene construction ability is associated with the ability to understand our own and other people’s mental states, and that these individual-level preferences/cognitive styles can overrule typical group-level characteristics.

Episodic memory describes the conscious recollection of personally experienced events (e.g. time, location, emotions and other contextual information). According to the multi-store model of memory (Atkinson & Shiffrin, 1968), episodic memories are encoded into and retrieved from long-term memory through a series of memory stores. First, information from the environment is perceived through our five senses (sight, sound, smell, touch and taste) and stored briefly (<1 s) in sensory memory. If the information is attended to, then it flows into short-term memory, which has a duration of 20 s, and a capacity of 7 ± 2 chunks (Miller, 1956). If this information is rehearsed, then it is encoded into long-term memory, which has an unlimited duration and capacity, and includes episodic, procedural (knowledge of how to do things) and semantic (general knowledge) memory. Information transfer is bi-directional; memories can be retrieved from long-term storage into short-term memory, and from there, can be brought into consciousness.

Episodic simulation is the ability to mentally simulate or imagine events (Schacter et al., 2012) and is therefore a key component of episodic memory (Maguire, 2001; Rugg et al., 2002; Svoboda et al., 2006), episodic future thinking (Addis et al., 2007; Atance & O’Neill, 2001; Szpunar et al., 2007), spatial navigation (Burgess et al., 2002), theory of mind (Frith & Frith, 2003) and ‘mind-wandering’ (Mason et al., 2007). The capacity for ‘self-projection’ – which is the ability to shift one’s current state of self into the past (episodic remembering) or future (episodic future thinking) to mentally simulate an alternative perspective (i.e. mental time travel of the self, or autonoetic consciousness; Tulving, 2005) – is thought to be a crucial common cognitive process that is associated with all of these skills (Buckner & Carroll, 2007). Episodic simulation and self-projection capacity appear to emerge in parallel during early development (e.g. Quon & Atance, 2010; Suddendorf, 2010), and are disrupted in some developmental disorders. In this article, we examine episodic simulation in adults with autism spectrum conditions (ASC),^1^ testing some of the sensory and self-referential features of these simulations, and exploring some of the cognitive predictors of scene construction success.

Research suggests that autistic individuals experience specific difficulties in recalling episodic memories (see Desaunay et al., 2023 for a review), and with episodic simulations of future events. For example, Lind and Bowler (2010; see also Lind et al., 2014a) observed that autistic adults produced significantly lower in quality (i.e. less specificity) verbal descriptions about specific events in their past or imagined events in the future than age- and IQ-matched neurotypical adults (medium effect size: *r* = 0.43). This suggests that self-projection capacity is diminished in autism. Furthermore, although past and future-oriented thinking descriptions were positively correlated in neurotypical participants (*r* = 0.72), they were not correlated in autistic participants (*r* = −.25). This might reflect the fact that autistic individuals draw less on episodic memory – phenomenological details such as feelings, emotions and sensory details stored in memory from past scenarios (e.g. last weekend at the beach, I got sun burnt) – to simulate or pre-experience a possible future scenario (e.g. next weekend at the beach, it will be sunny; Atance & O’Neill, 2005; Suddendorf & Corballis, 1997; Wheeler et al., 1997). Rather, they rely more on semantic memory – that is, general knowledge (e.g. memory for facts) – to imagine future events, and perhaps also to recall past events (e.g. Bon et al., 2013; Ciaramelli et al., 2018; Crane et al., 2012; Goddard et al., 2014; McDonnell et al., 2017; Terrett et al., 2013). Thus, difficulties with episodic future thinking and episodic memory (Bowler et al., 2011, 2014; Gaigg et al., 2015; Lind & Bowler, 2008) might be caused by a reduced capacity in aspects of self-awareness – specifically an awareness of one’s personal continuity through time – that is, temporally extended self-awareness (Lind & Bowler, 2010).

However, one way of imagining a *contextually rich* future scenario, is to extract episodic and contextual details from past episodes, and flexibly recombine them into new configurations. Using this method, episodic future thinking is dependent on the integrity of the episodic memory system (Irish et al., 2012). Thus, reduced temporally extended self-awareness might adversely affect the use of self-experience to construct and mentally project oneself through time to identify with an anticipated future or past state of self (Lind & Bowler, 2010; Lind, Bowler, & Raber, 2014). Therefore, reliance on semantic knowledge – which does not require self-projection – might be used to compensate for self-projection difficulties (Ciaramelli et al., 2018; Terret et al., 2013), but could diminish the ability to simulate *contextually rich* future events (e.g. Lind & Bowler, 2010).

## Scene construction

Scene construction is the ability to mentally generate (i.e. imagine) and maintain a coherent spatial scene/event in mind; it is therefore also critical for episodic simulation. It is typically operationalised by asking participants to imagine atemporal and impersonal fictitious scenes (e.g. ‘Imagine you’re lying on a beach in a tropical bay’), and asking them to ‘describe what you can see, hear, smell and feel in as much detail as possible’ (e.g. Hassabis et al., 2007). Using this approach, Lind et al. (2014b) found that autistic adults produced significantly lower quality descriptions for *both* episodic memories/ episodic future episodes which were self-relevant (i.e. measuring *self-projection*, e.g. ‘Imagine a possible future meeting that you might have with a friend’) and self-irrelevant (i.e. measuring *scene construction*, e.g. ‘Imagine you’re lying on a sandy beach in a tropical bay’). Lind et al.’s (2014b) observation of diminished *scene construction* ability in autistic relative to neurotypical adults (*N* = 56; large effect size, Cohen’s *d* = 0.85) despite being matched with the neurotypical group on verbal IQ, has been replicated by Black et al. (2018; *N* = 50). Importantly, this difficulty in autism was comparable to that observed for *self-projection* and was independent of general narrative ability (Lind et al., 2014b). Since scene construction does not require self-projection, this shows that diminished episodic simulation ability in autism cannot be attributed solely to difficulties in self-projection, and that reduced levels of scene construction might be the critical factor.

These findings of diminished scene construction ability map onto Bowler et al.’s (2011) relational binding account, which proposes that relational binding capacity is reduced in autism, characterised by difficulties linking elements of experience to one another and/or to their spatial and/or temporal context. This proposal echoes the notion of ‘weak central coherence’ in autism, which is a cognitive/perceptual processing style that is characterised by difficulty processing environmental stimuli as a coherent whole (global processing), instead focusing on each individual element (feature-based processing). The importance of relational binding for episodic simulation is supported by Gaigg et al. (2015) who observed that, in both neurotypical and autistic adults, contextually rich (episodic) recollection (i.e. *remembering*) of word triads increased with the number of category relations shared between words; this was not observed for familiarity-based (semantic) retrieval (i.e. *knowing*). This suggests that category relations support contextually rich recollection in both neurotypical and autistic participants. So, under real-world conditions, when individuals are required to form and store associations between items themselves, the less contextually rich recollection in autism – that is, relying less on episodic *remembering* and more on semantic *knowing* (e.g. Lind & Bowler, 2010) – might reflect a reduced capacity to form associations between items at encoding. Consequently, the ability to flexibly retrieve multisensory information as an integrated whole and mentally simulate a coherent episode is diminished. In this article, we use a scene construction task (Hassabis et al., 2007) to compare episodic simulation capacity in a large sample of autistic and neurotypical adults (*N* = 118), and examine whether differences exist in sensory experiences and self-referential cognition in these mental simulations.

## Sensory experiences

According to Viberg (1983), in neurotypical people the frequency of sense modalities in spontaneous conversation follows a hierarchy of sight > sound > touch > taste > smell. However, sensory perception is estimated to be atypical in 69%–100% of autistic people (e.g. Baranek et al., 2006; Hilton et al., 2010; Leekam et al., 2007), characterised by hyper- and/or hypo- attention, perception, memory, etc. This atypicality is explained by the intense world theory (Markram & Markram, 2010) in terms of hyperactivation of brain circuits, which causes inefficient multisensory integration (Brock et al., 2002; Foss-Feig et al., 2010; Waterhouse et al., 1996). The ‘predictive coding theory’ of autism (van Boxtel & Lu, 2013; Van de Cruys et al., 2014) proposes that diminished meta-learning abilities cause difficulty distinguishing between important and less important prediction errors. Consequently, autistic people struggle to contextualise incoming information and so struggle to make appropriate predictions based on previous experience. Therefore, an extended temporal bindinWangg window in autistic *versus* neurotypical people might create a ‘fuzzier’ sensory environment (Wallace & Stevenson, 2014) in which unrelated stimuli become integrated (McGurk & MacDonald, 1976; Shams et al., 2000; Stevenson et al., 2012; Thye et al., 2018).

If level of involvement of a sensory experience (such as sound, touch, smell, taste, sight) predicts the likelihood of it being encoded (e.g. hyper-perception of sound increases the probability of it being encoded), then a ‘fuzzier’ sensory environment in autism might predict that descriptions of episodic simulation have atypical reliance on sensory modalities (Wallace & Stevenson, 2014). Indeed, Anger et al. (2019) observed that when asked to describe episodic memories and episodic future thinking in as much multimodal detail as possible, autistic adolescents (M_age_ = 13.5 years) produced significantly lower quality descriptions – including fewer and lower intensity sensory details (colour, smell, sound and tactile feeling) – than matched neurotypical adolescents during *free recall*. Nevertheless, autistic adolescents performed comparably to neurotypical adolescents when provided with visual cues that prompted for details such as what, how when, where, who, emotions, Likert-type scales, perceptions (e.g. colours) and perspective (allocentric vs egocentric). This suggests that visual scaffolding can support autistic individuals to produce coherent and detailed narratives (see also Mattison et al., 2015, 2018).

One suggestion is that the underlying cognitive mechanisms that allow processing and storing of relevant sensory information (bottom-up process) are preserved in autism, but the ability to use prior experience and context to *efficiently* understand and integrate/modulate such sensory/contextual information (top-down process) for social/narrative purposes is impaired (Bowler et al., 2004). This means that the narration of episodic events can appear disrupted due to difficulties knowing what social/sensory information to provide (e.g. Hamilton, 2013; Southgate & Hamilton, 2008; Wang & Hamilton, 2012). To reduce the influence of ambiguous task demands in our task, we explicitly prompted participants to include sensory details in their scene construction descriptions, compared the relative mention of sensory experiences in the five subdomains, and tested the degree to which any differences in episodic scene construction and sensory experience could be attributed to individual differences in cognition (including ToM).

## Self-referential cognition

Among neurotypical individuals, a *self-bias* or ‘self-reference effect’ is commonly observed, which is characterised by preferential processing of self-relevant information relative to other-relevant information (Tversky & Kahneman, 1974). In ASC, self-bias has been found to be reduced or absent in some contexts (Burrows et al., 2017; Grisdale et al., 2014; Henderson et al., 2009; Lombardo et al., 2007), suggesting that self-referential cognition is diminished relative to neurotypical people. However, self-bias has been found to be intact in autistic adults and children in the domains of perception/attention (Williams et al., 2018) and memory (Lind et al., 2020), and seems to be unrelated to the number of autistic traits (Lind et al., 2020; Williams et al., 2018). Furthermore, research suggests that autistic people do not have diminished ability to imagine the self in the episodic future, but might have difficulty in the episodic past (Lind & Bowler, 2010). This contradictory evidence suggests that self-biases might operate differently across different cognitive domains, thus further research is needed to fully understand this area.

Self-referential cognition is likely to have important implications for scene construction, however its role in episodic future thinking has not been thoroughly examined to date. The neural-level model of spatial memory and imagery (Bicanski & Burgess, 2018; Burgess et al., 2001; Byrne et al., 2007) proposes that *egocentric* (i.e. body-centred; viewpoint-dependent) representations of the environment – which uses the self as a reference and corresponds to a specific point of view (i.e. head/gaze direction) – are transformed into *allocentric* (i.e. world-centred; viewpoint-independent) representations. Allocentric representations are dependent on processing the relations between landmarks independent of a single view-point. The reverse process allows reconstruction of egocentric representations from stored allocentric representations. If we extract episodic and contextual details from past episodes to construct a *contextually rich* (i.e. episodic) fictitious scene, then one must go beyond the *allocentric* representations stored in memory and use these to construct an *egocentric* representation. In this article, we examine whether self-referential cognition is diminished in autism by testing participants’ use of the egocentric perspective in scene construction – indexed by first-person pronoun use (e.g. I, me, my, mine, we, our).

## Present study and hypotheses

The current study aimed to investigate scene construction ability in a larger sample of autistic adults and age- and IQ-matched neurotypical adults than previous studies have used. Moreover, it advances previous research on episodic simulations in autism by analysing the nature of sensory experiences and self-reference in this scene construction task. We also explored some of the cognitive predictors of successful scene construction, including autistic traits, ToM and alexithymia (which is characterised by difficulty identifying and describing one’s own and others’ emotions). Our hypotheses were as follows:

Hypothesis 1a (H1a). Based on findings from Lind et al. (2014b) and Black et al. (2018), we predicted a generally diminished scene construction ability in autistic adults compared to neurotypical adults.
*Hypothesis 1b (H1b).* We predicted that several variables would correlate with scene construction ability. Specifically, we expected that participants with a higher number of autistic traits (measured on the AQ and ADOS), higher alexithymia (TAS-20 scale), and lower ToM ability (animations task) would show a reduced scene construction ability.
*Hypothesis 2a (H2a).* We predicted that sensory experiences would show a general hierarchy of mention: sight > sound > touch > taste = smell. Moreover, based on Anger et al. (2019) and the free recall nature of the scene construction task, we predicted that autistic adults would show reduced sensory experiences compared to neurotypical adults.
*Hypothesis 2b (H2b).* We predicted that several variables would correlate with frequency of sensory experience. Specifically, we expected that participants with higher number of autistic traits (measured on the AQ and ADOS), higher alexithymia (TAS-20 scale), lower ToM ability (animations task), and lower experiential index, would have lower frequency of sensory experience.
*Hypothesis 3a (H3a).* Based on previous empirical evidence on self-bias (Burrows et al., 2017; Grisdale et al., 2014; Henderson et al., 2009; Lombardo et al., 2007) we predicted that self-reference would be reduced in autistic adults compared to neurotypical adults.
*Hypothesis 3b (H3b).* We predicted that several variables would correlate with self-reference. Specifically, we expected that increased frequency of self-reference would be associated with a higher experiential index score, sensory experience and ToM (animations score), and lower alexithymia (TAS-20 score). In contrast, we did not expect a significant correlation between self-reference frequency and autistic traits (AQ and ADOS).

## Method

### Participants

The data were collected as part of a battery of tasks given to participants in the department’s Autism database and included 63 (41 male; 22 female) neurotypical adults with no history of psychiatric or neurological conditions (self-report) and 55 (37 male; 18 female) adults with a clinical diagnosis of ASC, of which official diagnostic information was checked. An a priori power analysis using G*Power (Faul et al., 2007) revealed that 16 participants would be enough to detect a main effect of group on the experiential index score, with effect size Cohen’s *d* = 0.85 (Black et al., 2018) and power of 0.90. All participants self-reported as native English speakers. Participant groups were matched on age, gender, and Intelligence Quotient (IQ, Wechsler Adult Intelligence Scale, WAIS-III or WAIS-IV; Wechsler, 1997, 2008), but were not matched on Autism spectrum Quotient (AQ; Baron-Cohen et al., 2001) (see Table 1). Current autistic characteristics were assessed for all autistic participants using module 4 of the Autism Diagnostic Observation Schedule (ADOS; Lord et al., 2000; see Supplementary Materials), and videos were double coded to ensure reliability of scoring (inter-rater reliability was found to be excellent with intraclass correlation of 0.89). In the autistic group, 26 out of the 51 who completed the ADOS met the clinical cut-off (i.e. a score of 7 or above), indicating significant autistic traits.^2^

**Table 1.** Participant characteristics and statistical comparisons between diagnostic groups (autistic/neurotypical).

	Neurotypical	ASC	Neurotypical	ASC	*t*	*df*	*p*	*d*	BF^10^
	*N*	*N*	Mean (*SD*)	Mean (*SD*)					
Age (years)	63	55	31.52 (10.27)	30.50 (11.52)	0.51	116	.61	0.09	0.22
IQ: Full Scale	63	54	105.29 (10.41)	103.20 (15.01)	0.88	115	.38	0.16	0.28
IQ: Verbal	63	54	103.02 (10.75)	102.81 (12.20)	0.10	115	.93	0.02	0.20
IQ: Performance	63	54	106.56 (12.83)	103.37 (18.61)	1.09	115	.28	0.20	0.34
AQ	62	55	17.08 (6.2)	31.00 (8.80)	10.00	115	<.001***	1.85	>100
ADOS	-	51	—	7.27 (4.67)	—	—	—	—	—
Animations	22	32	5.55 (1.90)	4.56 (2.17)	1.72	52	.046*	0.48	0.93
TAS-20	31	40	45.55 (9.41)	59.08 (12.53)	5.01	69	<.001***	1.19	>100

ASC: autism spectrum conditions; IQ: Intelligence Quotient; AQ: Autism-spectrum Quotient; ADOS: Autism Diagnostic Observation Schedule; TAS: Toronto Alexithymia Scale.

Some tasks have missing data, so statistics are reported on maximal data for each task.

* *p* < 0.05; ***p < 0.001.

### Materials and procedure

Full details of measures, scoring and criteria for significance are provided in Supplementary Materials.

#### Scene construction task

The scene construction task, described by Hassabis et al. (2007), measured the ability to mentally construct a unique/novel visual scene. The experimenter asked the participant to close their eyes and vividly imagine three ordinary fictitious scenes (e.g. ‘Imagine you’re lying on beach in a tropical bay’). They were then given 2 to 3 min to ‘Describe what you can see, hear, smell and feel in as much detail as possible’. Importantly, participants were asked not to recount an actual memory of an experienced event, or something they planned to do, but rather, to create something new. As outlined in Hassabis et al. (2007), a probing protocol was followed whereby *general* prompts were given if a description could not be provided or lacked detail (e.g. ‘Tell me more about this scene’). Participants were probed to move on if they became fixated on a particular aspect of a scene and were probed to elaborate further if they provided only poor detail. They were encouraged to continue with their descriptions until their account concluded or they were unable to elaborate further.

*Experiential Index Score*: Transcriptions of verbal descriptions are available for all participants on our OSF page (https://osf.io/qdyvk/).^3^ As in Hassabis et al. (2007, and detailed in Supplementary Materials), verbal descriptions were coded for spatial coherence, content and quality, and combined with participant ratings of the imagined scene to provide an imagination index (an ‘experiential index score’), ranging from 0 (not experienced at all) to 60 (extremely richly experienced), with higher scores indicating greater imagination ability. Five percent of the transcriptions were second-coded by the second author to check the inter-rater reliability of the coding. Substantial inter-rater reliability was found, with intraclass correlation of 0.89, *F*(14, 14) = 11.42, *p* < 0.001.

In addition to the main imagination criteria based on existing coding of the data, we defined two additional coding criteria to address our specific questions.

*Self-Related Centre of Reference*: Within each scenario description, every reference to a first-person pronoun (e.g. ‘I, me, my, mine, myself, we, us, our’) was scored one point, and a sum total of self-references was calculated for each scenario, then averaged across the three scenarios for each participant.

*Sensory Elements*: Within each scenario description, every reference to a sensory element (sight/sound/taste/touch/smell, e.g. touch: ‘It felt warm’) was scored one point. This was calculated separately for each of the five key sensory modalities (sight/touch/sound/taste/smell). To avoid ceiling effects on *sight* references, simply mentioning an entity (e.g. ‘I could see the sea’) was not awarded any points. Rather, only statements describing the properties of an entity were scored one point (e.g. ‘the sea was blue and green’). Transcriptions (5%) were second-coded by the second author to check the inter-rater reliability of the coding. Almost perfect inter-rater reliability was found, with intraclass correlation of 0.97 (*F*(19, 19) = 35.49, *p* < 0.001).

#### Measures of theory of mind, autistic traits and emotions

Three established measures were used to assess Theory of Mind (Animations Task; Abell et al., 2000), Autistic traits (Autism-spectrum Quotient (AQ); Baron-Cohen et al., 2001) and alexithymia (the ability to identify and describe emotions; Toronto Alexithymia Scale,^4^ TAS-20; Bagby et al., 1994); see Supplementary Materials.

#### Community involvement

There was no community involvement in the design or implementation of the reported study.

## Results

The datasets and transcripts of verbal descriptions in the scene construction task are available on the OSF project page (https://osf.io/qdyvk/).

First, we ran analyses to test whether the length (number of words) of scene construction descriptions and number of prompts given by the experimenter were comparable across the two diagnosis groups. Length did not differ between groups, *t*(116) = 1.59, *p* = 0.12, but there were significantly more prompts to autistic (*M* = 0.55, *SD* = 1.15) than neurotypical (*M* = 0.10, *SD* = 0.43) participants, *t*(116) = 2.88; *p* = 0.01. Second, we identified and excluded statistically significant outliers – that is, those that were more than three standard deviations away from the overall sample mean – on the experiential index, sensory experience, and/or self-reference measures. This procedure identified two participants from the ASC group with a frequency of self-reference that was substantially greater than 3 standard deviations from the sample mean (75.67 and 68.67 vs *M* = 8.78 for the whole group) and these participants were excluded from further analysis on this measure.

### How does autism diagnosis influence scene construction ability?

**Figure 1.**
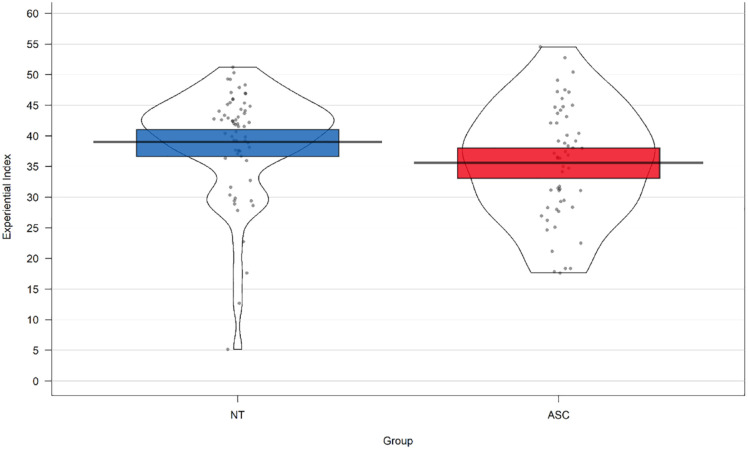
Mean experiential index score in each diagnostic group.

The experiential index score was normally distributed in the autistic group, W(55) = 0.98, *p* = 0.54, but showed a negatively skewed distribution in the neurotypical group, W(63) = 0.86, *p* = < 0.001. Therefore, a Kruskal–Wallis H non-parametric test was used to test the difference in experiential index between autistic and neurotypical groups (see Figure 1). This revealed a significantly higher mean rank experiential index score in the neurotypical (*M* = 66.71) than the ASC group (*M* = 51.25), χ^2^(1) = 6.00, *p* = 0.014.

### What are the cognitive predictors of scene construction ability?

We ran correlation analyses to test the relations between experiential index and (1) AQ, (2) animations, (3) TAS-20, and (4) ADOS total (ASC group only). Since the categorical analysis observed a significant main effect of diagnosis on experiential index, we ran separate exploratory correlations for the autistic and neurotypical groups (see Table 2).

**Table 2.** Matrix displaying correlations between experiential index and cognitive predictors in the autistic and neurotypical groups separately.

		Experiential index	AQ	Animations	ADOS
Autistic					
AQ (*N* = 55)	Pearson’s r	–.041	–		
	BF^10^	0.176			
Animations (*N* = 32)	Pearson’s r	.373*	–.009	–	
	BF^10^	1.816	0.220		
TAS-20 (*N* = 40)	Pearson’s r	–.220	.589**	.173	–.064
	BF^10^	0.482	> 100	0.338	0.212
ADOS (*N* = 51)	Pearson’s r	–.242	–.061	–.255	
	BF^10^	0.731	0.191	0.569	
Neurotypical					
AQ (*N* = 62)	Pearson’s r	–.298*	–		
	BF^10^	2.378			
Animations (*N* = 22)	Pearson’s r	.353	.191	–	
	BF^10^	0.897	0.373		
TAS-20 (*N* = 40)	Pearson’s r	–.312	.468**	.408	
	BF^10^	0.902	5.784	1.234	

AQ: Autism-spectrum Quotient; ADOS: Autism Diagnostic Observation Schedule; TAS: Toronto Alexithymia Scale.

* *p* < 0.05; **p < 0.01; *** *p* < 0.001.

In the autistic group analysis, there was a significant positive correlation between experiential index and animations score, *r*(32) = 0.37, *p* = 0.04, BF^10^ = 1.82; participants who scored higher on the experiential index also scored higher on the animations task. In the neurotypical group analysis, there was a significant negative correlation between experiential index and AQ, *r*(62) = -.30, *p* = 0.02, BF^10^ = 2.38; participants who scored higher on the experiential index scored lower on the AQ scale.

Next, a multiple regression analysis was conducted across the whole group of participants,^5^ to test whether experiential index score was predicted by individual performance on the animations task, or AQ and TAS-20 scales (these three variables were included because they were significantly correlated with experiential index). In addition, the diagnostic group was included in the regression model. This regression analysis revealed that performance on the animations and TAS-20 were significant predictors of participants’ experiential index (although the Bayes factors suggest that these represent only anecdotal evidence), but AQ and diagnosis did not significantly predict the experiential index once these effects were accounted for (see Table 3).

**Table 3.** Multiple regression analysis results summary.

	*SE B*	*β*	95% CI *B*	*p*
Total sample: Experiential index; *R*^2^ Adj = 0.20, *F*(4, 45) = 4.12, *p* = 0.006**				
Animations	.657	.446	.786, 3.435	.002***
TAS-20	.145	–.474	–.634, –.048	.024*
AQ	.252	.172	–.337, 0.680	.501
Diagnosis	3.743	–.057	–8.736, 6.351	.751

CI: Confidence interval; TAS: Toronto Alexithymia Scale; AQ: Autism-spectrum Quotient.

* *p* < 0.05; **p < 0.01; ***p < 0.001.

### How does autism diagnosis influence scene construction of sensory experiences?

**Figure 2.**
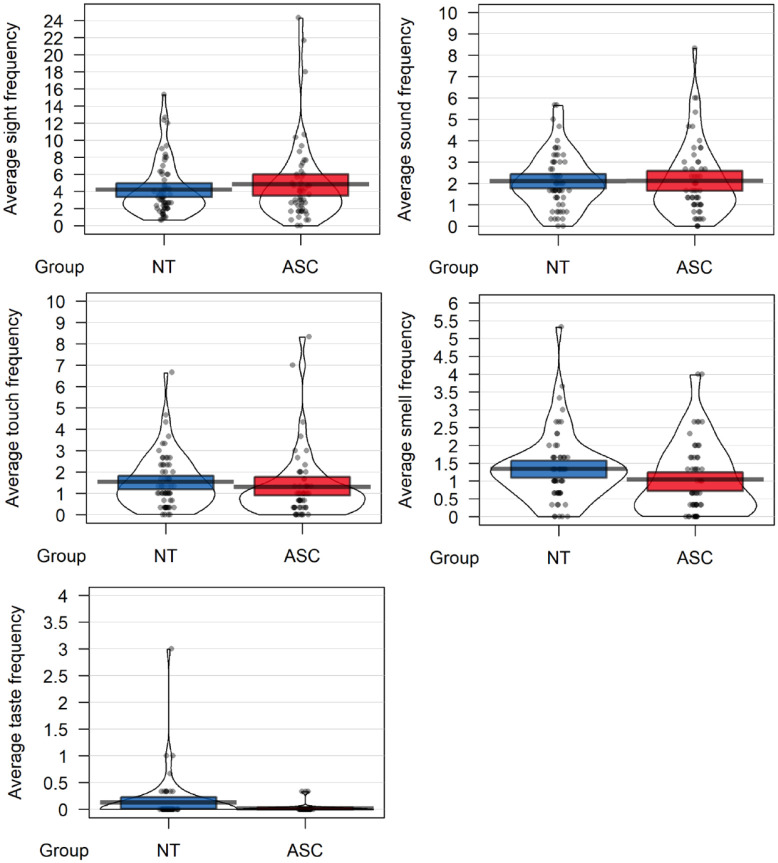
Mean frequency of sensory descriptions for each sensory modality and diagnostic group.

To investigate the effect of diagnosis and sensory modality on sensory descriptions, a 2 (Diagnosis: ASC/NT) × 5 (Sensory Modality: sound/taste/touch/smell/sight) mixed ANOVA was conducted on the frequency of sensory references, with repeated measures on the last factor (see Figure 2). This revealed a significant main effect of sensory modality, *F*(4, 464) = 99.24, *p* < 0.001, *η*_p_^2^ = 0.46, BF^10^ > 100. A series of paired samples *t*-tests revealed that the frequency of sensory references for each sensory modality followed a pattern of sight > sound > touch = smell > taste. Sight was referenced significantly more that sound, *t*(117) = −6.94, *p* < 0.001, *d* = −.64. Sound was referenced significantly more than touch, *t*(117) = −4.51, *p* < 0.001, *d* = −.42, and smell, *t*(117) = 6.95, *p* < 0.001, *d* = −.64. Reference to smell and touch did not significantly differ in frequency, *t*(117) = −1.94, *p* = 0.054, *d* = -.18. Taste was referenced significantly less than both smell, *t*(117) = 12.56, *p* < 0.001, *d* = -1.16, and touch, *t*(117) = 12.56, *p* < 0.001, *d* = 1.16. There was no main effect of diagnosis, *F*(1,116) < 0.01, *p* = 0.99, *η*_p_^2^ < 0.001, BF^10^ = 0.14, and no diagnosis × sensory modality interaction, *F*(4, 464) = 1.2, *p* = 0.31, *η*_p_^2^ = 0.01, BF^10^ = < 0.001. Exploratory comparisons between groups for each sensory modality also revealed no difference in frequency between groups.

### What are the cognitive predictors of sensory scene construction?

Correlation analyses investigating the relations between sensory experience (summed frequency across all five senses) and (1) experiential index, (2) AQ, (3) animations, and (4) TAS-20, was conducted across the whole group of participants since group did not modulate effects in the categorical analysis (see Table 4).

**Table 4.** Matrix displaying correlations between sensory experience and cognitive predictors in the total sample.

		Sensory experience	Experiential index	AQ	Animations
Total sample					
Experiential index (*N* = 118)	Pearson’s r	.629**			
	BF^10^	> 100			
AQ (*N* = 117)	Pearson’s *r*	–.026	–.233*		
	BF^10^	0.120	2.679		
Animations (*N* = 54)	Pearson’s *r*	.394**	.399**	–.134	
	BF^10^	11.667	13.00	0.267	
TAS-20 (*N* = 71)	Pearson’s *r*	–.062	–.292*	.690**	.084
	BF^10^	0.168	3.00	> 100	0.206

AQ: Autism-spectrum Quotient; TAS: Toronto Alexithymia Scale.

* *p* < 0.05; **p < 0.01; ***p < 0.001.

Analysis revealed a significant positive correlation between frequency of sensory experience and experiential index, *r*(118) = 0.63, *p* < 0.001, BF^10^ = 19.88, and animations score, *r*(54) = 0.39, *p* = 0.003, BF^10^ = 11.67, whereby participants with a higher frequency of sensory references also scored higher on the experiential index and the animations task.

### How does autism diagnosis influence self-referential scene construction?

**Figure 3.**
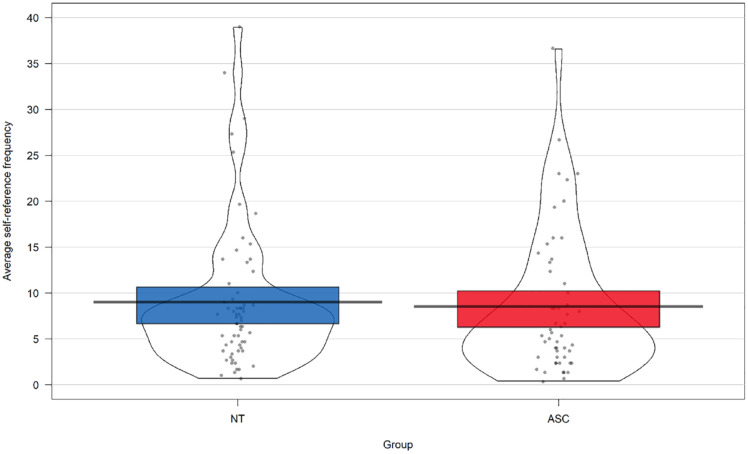
Mean self-reference frequency in each diagnostic group.

To investigate the effect of diagnosis on self-reference during scene construction, a one-way between subjects (Diagnosis: ASC/NT) ANOVA was conducted on frequency of first-person pronoun use (see Figure 3). There was no significant main effect of diagnosis, *F*(1, 114) = 0.11, *p* = 0.74, *η*_p_^2^ = 0.001, BF^10^ = 0.21, thus participant groups did not differ in their likelihood of self-reference.^6^

### What are the cognitive predictors of self-referential scene construction?

Since the previous categorical analysis observed no significant main effect of diagnosis, the subsequent correlation analyses tested associations between self-reference and (1) experiential index, (2) sensory experience, (3) AQ, (4) animations, and (5) TAS-20, across the whole group of participants (see Table 5).

**Table 5.** Matrix displaying correlations between self-reference and cognitive predictors in the total sample.

		Self-reference	Experiential index	Sensory experience	AQ	Animations
Total sample						
Experiential index (*N* = 116)	Pearson’s *r*	.361**	–			
	BF^10^	> 100				
Sensory experience (*N* = 116)	Pearson’s *r*	.329**	.629**	–		
	BF^10^	70.069	> 100			
AQ (*N* = 115)	Pearson’s *r*	−.017	−.233*	−.026	–	
	BF^10^	0.118	2.679	0.120		
Animations (*N* = 54)	Pearson’s *r*	.116	.399**	.394**	−.134	–
	BF^10^	0.238	12.997	11.667	0.267	
TAS-20 (*N* = 71)	Pearson’s *r*	−.058	−.292*	−.062	.690**	.084
	BF^10^	0.166	2.966	0.168	>100	0.206

AQ: Autism-spectrum Quotient; TAS: Toronto Alexithymia Scale.

* *p* < 0.05; **p < 0.01; ***p < 0.001.

Self-reference was positively correlated with experiential index, *r*(116) = 0.54, *p* < 0.001, BF^10^ ⩾ 100, and sensory experience, *r*(116) = 0.33, *p* < 0.001, BF^10^ ⩾ 100, whereby participants who used more frequent first-person pronouns scored higher on the experiential index score and described more sensory experiences.

## Discussion

This study aimed to investigate scene construction ability in a larger sample of autistic and neurotypical adults than previous studies. Moreover, we advanced previous research on scene construction in autism by analysing participants’ sensory experiences and self-reference and ran exploratory analyses on some of the cognitive predictors of scene construction ability (including ToM, autistic traits, and alexithymia). Hassabis et al.’s (2007) scene construction task was used, in which participants were asked to vividly imagine and describe fictitious scenes; descriptions were also coded for frequency of first-person pronouns and sensory experiences.

### Scene construction

Scene construction ability was significantly better – characterised by descriptions that were greater in quality (i.e. more specific, more episodic, more coherent) – in neurotypical than autistic participants. Furthermore, scene construction ability was negatively associated with autistic traits (AQ): participants with a higher experiential index had fewer autistic traits than participants with a lower experiential index. This pattern replicates that reported in previous studies that have used smaller participant samples (Black et al., 2018; Lind et al., 2014b), thus increasing our confidence in the reliability of these findings. According to Hassabis et al. (2007), episodic simulation of atemporal, fictitious scenes which are not self-relevant does not require self-projection. This suggests that episodic simulation ability in autistic adults cannot be attributed solely to difficulties in self-projection, and that reduced capacity for scene construction might be the critical factor (Lind et al., 2014b). Diminished scene construction ability in autism might be caused by diminished hippocampally-mediated *relational binding* (Bowler et al., 2011) or ‘weak central coherence’ (WCC), which causes diminished multisensory integration of phenomenological details (e.g. Foss-Feig et al., 2010; Waterhouse et al., 1996). Consequently, this might reduce the ability to flexibly retrieve this multisensory information as an integrated whole, required to mentally construct a coherent scene in mind (Lind et al., 2014b; Mullally & Maguire, 2014).

As predicted, scene construction ability was significantly associated with ToM (animations task) in the autistic group, and with autistic traits (AQ) in the NT group: participants who had greater scene construction ability (a higher experiential index) had better ToM ability (higher animations score) and fewer autistic traits (lower AQ score). These findings are in line with the notion that scene construction is commonly associated with a series of cognitive functions – including the ability to understand one’s own and others’ mental states (i.e. good ToM) – that involve episodic simulation (Schacter et al., 2012).^7^ For example, neuroimaging evidence has suggested that brain regions typically associated with ToM are active during scene construction, although significantly less than during self-related episodic future thinking and episodic remembering (see Hassabis et al., 2007). We note that our findings are limited by the relatively small battery of cognitive predictors we tested; it is likely that other variables, not measured here, contribute to the pattern of results reported here (e.g. episodic memory, general knowledge and age, Schacter et al., 2012).

A multiple regression analysis further explored the cognitive predictors of scene construction ability in the total sample, and revealed that when diagnosis and autistic traits (AQ) were included in a multiple regression model along with ToM (animations score) and alexithymia (TAS-20 score) measures, diagnosis and autistic traits no longer predicted scene construction ability, but ToM and alexithymia both remained significant predictors. Since these cognitive characteristics are highly prevalent in, and key characteristics of, ASC (Baron-Cohen, 1989; Baron-Cohen et al., 1985; Berthoz & Hill, 2005; Castelli, 2002; Deschrijver et al., 2016; Salminen et al., 1999; Tager-Flusberg, 2007), it is not surprising that diagnosis and autistic traits drop out when these cognitive characteristics are included as predictors. This suggests that the less coherent, and more fragmented scene construction descriptions given by the autistic group might not be associated with ASC specifically – that is, impaired relational binding – but might be related to diagnosis-defining social difficulties including alexithymia and ToM impairment (or the underlying cognitive mechanisms associated with ToM and alexithymia), which are both more prevalent in ASC than in the neurotypical population. As such, neurotypical individuals with poor scene construction ability are expected to show similar levels of alexithymia and difficulties with ToM as autistic individuals, though this individual cognitive profile differs from the typical group-level characteristics of neurotypical people.

### Sensory experiences

To investigate the relation between scene construction and perceptual processing style, the current study investigated the frequency of sensory references in each of the five key sensory modalities (sound, smell, sight, touch, taste). This is the first time that sensory episodic experiences in scene construction have been compared between autistic and neurotypical groups. Across participant groups, the frequency of sensory references for each sensory modality followed a pattern of sight > sound > touch = smell > taste, showing that some sensory modalities were more frequently used to support scene construction than other sensory modalities (Viberg, 1983). Importantly, the frequency of sensory reference did not differ according to diagnosis and was not associated with autistic traits. This suggests that diminished scene construction ability is not influenced by atypical sensory experience.

However, it might be that the explicit prompt to include sensory details in descriptions provided sufficient task support to enable typical performance in the ASC group. Previous research found that autistic participants’ episodic descriptions were similar to neurotypical participants *only* when provided with task support cueing what information to provide (i.e. pictures cueing for components: what, how when, where, who, emotions, Likert-type scales, perceptions (e.g. colours); perspective (allocentric vs egocentric) etc.) Anger et al. (2019). Therefore, it is possible that one of the limiting factors in scene construction ability in ASC, and particularly in detailing a sufficiently rich sensory experience, is ambiguity in understanding what social/sensory information to provide (e.g. Hamilton, 2013; Southgate & Hamilton, 2008; Wang & Hamilton, 2012). Supporting this, in the current study, ToM (animations) was positively associated with sensory descriptions, whereby participants with higher ToM ability – and hence better understanding of social/narrative conventions – provided more descriptions of sensory experiences. Furthermore, the frequency of sensory reference was positively correlated with scene construction ability (experiential index), reflecting the overlap in what is captured by these measures. This is in line with our suggestion that ToM might contribute to better understanding of social narrative conventions including sensory processing, likely due to better language skills or better flexibility in processing, which in turn supports scene construction ability.

### Self-referential cognition

This is also the first time that frequency of self-reference in scene construction has been compared between autistic and neurotypical groups. Contrary to our predictions, the frequency of self-reference did not differ according to diagnosis, and was not associated with autistic traits (AQ and ADOS). This challenges the notion that self-referential cognition is routinely absent/diminished in ASC, and instead appears in line with more recent evidence suggesting that self-referential cognition is intact in some tasks/cognitive domains (Lind et al., 2020; Williams et al., 2018). Intact frequency of self-reference suggests that autistic participants appropriately reconstructed egocentric representations from stored allocentric representations. Therefore, it might be that difficulties are specific to episodic memory (Lind & Bowler, 2010).

Furthermore, this frequency of self-reference (egocentric perspective) was positively correlated with scene construction ability (experiential index) and sensory experience: participants with higher frequency of self-reference, had enhanced scene construction ability and a higher frequency of sensory experiences. This suggests that participants appropriately used episodic memory of past experiences to support construction of a contextually rich novel scene (Committeri et al., 2020). The ability to construct a contextually rich novel scene and episodic memory might both result from a higher-level capacity for flexible re-combinative processing. Contrary to our predictions, self-reference was not correlated with ToM (animations) ability, which suggests that self-referential processing does not consistently rely upon an understanding of narrative conventions.

## Conclusion

Overall, the current study supports previous research finding that scene construction ability is diminished in autistic relative to neurotypical adults (Black et al., 2018; Lind et al., 2014b). This suggests that episodic simulation ability cannot be attributed solely to difficulties in self-projection in ASC, and that diminished scene construction ability might be the critical factor (Lind et al., 2014b). We also explored some of the cognitive predictors of scene construction ability–the ability to infer others’ mental states (i.e. ToM) and describe emotions in themselves (i.e. alexithymia) – and found that individual differences in these are more closely associated with scene construction ability than group-level autism diagnosis. The current study further advances previous research by showing that the frequency of sensory experience and self-reference – both of which are thought to enhance scene construction descriptions – did not significantly differ according to diagnosis, and that sensory experience was positively correlated with ToM ability. This suggests that autistic adults can use sensory experiences and self-reference appropriately to support scene construction, though this (at least sensory experiences) might be dependent on good understanding of social-narrative conventions.

## Supplemental Material

sj-docx-1-aut-10.1177_13623613231216052 – Supplemental material for Scene construction ability in neurotypical and autistic adults Supplemental material, sj-docx-1-aut-10.1177_13623613231216052 for Scene construction ability in neurotypical and autistic adults by Marchella Smith, Lindsey Cameron and Heather J Ferguson in Autism

## Notes

1. We use a mixture of commonly endorsed terms to reflect the fact that ‘there is no single way of describing autism that is universally accepted and preferred’ (Kenny et al., 2016).

2. We note that while the ADOS total score is widely used as a ‘gold standard’ measure of ASC severity, it is influenced by chronological age, language aptitude and co-occurring clinical conditions (Gotham et al., 2009).

3. The transcripts should only be analysed and reported in publications with advance permission from Heather Ferguson (h.ferguson@kent.ac.uk), and citing this research paper/OSF repository.

4. While the main measures of experiential index scores, sensory experiences, and self-reference were coded and analysed by the authors from raw transcribed data, the predictor variables were collected from a database of pre-existing data, which includes summary statistics but not item-level responses for all participants. As such, while we recognise that there is a more up to date method to check the reliability of the TAS-20 score (see Z. J. Williams & Gotham, 2021), we are not able to use this method on the current data.

5. Note that some tests have missing data, so results are based on participants who have data for all variables (*N* = 49).

6. The main effect of diagnosis was also non-significant if the two outlier participants were included in the analysis *F*(1, 116) = 0.08, *p* = 0.77, *η*_p_^2^ = 0.001, BF^10^ = 0.28.

7. Importantly, the direction of causation – that is, whether diminished ToM ability cause diminished scene construction ability, *or* vice versa – cannot be determined from a correlation; therefore, this simply highlights the close link between the variables.
