# Supplementary material for: Scene construction ability in neurotypical and autistic adults
Source: Autism. 2023 Dec 28;28(8):1919–33. doi: 10.1177/13623613231216052 (PMC11301963; doi:10.1177/13623613231216052)
Supplement: sj-docx-1-aut-10.1177_13623613231216052 – Supplemental material for Scene construction ability in neurotypical and autistic adults [file sj-docx-1-aut-10.1177_13623613231216052.docx]

Scene Construction Ability in Neurotypical and Autistic Adults

**Supplementary Material**

**Participants**

Autistic participants had a clinical diagnosis of either autistic disorder (N=16), autism spectrum disorder (N=3), Asperger’s syndrome (N=26), Pervasive Developmental Disorder-Not Otherwise Specified (N=1), or not specified (N=9). Current autistic characteristics were assessed for all autistic participants using module 4 of the Autism Diagnostic Observation Schedule (ADOS; Lord et al., 2000). by a trained, research-reliable researcher. Videos were double coded to ensure reliability of scoring (inter-rater reliability was found to be excellent with intraclass correlation of .89).

**Scene Construction Task**

*Participant Ratings*: After each description, participants were asked to complete a questionnaire rating their imagined scenes for perceived salience/vividness (salience of the imager: 1, not vivid, to 5, very vivid), and for sense of presence/difficulty (how hard was the trial: 1, easy, to 5, hard).

*Spatial Coherence Index:* Participants were given a post-description questionnaire consisting of 12 statements, each providing a possible qualitative description of the newly constructed imagined scene/episode. Participants were asked to tick any of the statements that they thought accurately applied to their imagined scene/episode. Eight of the statements were ‘integrated’ and suggested that aspects of the constructed scene were contiguous (e.g., ‘I could see the whole scene in my mind’s eye’), and four of the statements were ‘fragmented’ and suggested that aspects of the constructed scene were not contiguous (e.g., ‘It was a collection of separate images’). For each integrated statement that was selected, one point was awarded, and each fragmented statement that was selected, one point was subtracted. This gave a score between -4 and +8, which was then normalised around zero to give a final spatial coherence index between -6 (completely fragmented) and +6 (completely integrated).

*Content*: The recordings of each scenario description were transcribed and blind coded by a trained experimenter. Within each scenario description, statements were coded as belonging to one of four main categories: spatial reference, entity presence, sensory description, or thought/emotion/action. Repeated statements, and other tangential information that could not be coded as belonging to one of the four categories were omitted. Hassabis et al. (2007) identified that seven details per category should be considered optimal performance. Therefore, each category has a maximum possible score of seven, which yielded a maximum possible content score of 28 for each scenario description. 10% of the transcriptions were also coded by a second author to check the inter-rater reliability of the coding.

*Quality Judgement*: Each scenario description was rated by the researcher for “how well they felt the description evoked a detailed ‘picture’ of the experience in their own mind’s eye” on a scale of 0 (no picture at all) to 10 (vivid, extremely rich picture).

*Experiential Index Score*: All the description and questionnaire scores detailed above were combined to provide an imagination index (an “experiential index score”), ranging from 0 (not experienced at all) to 60 (extremely richly experienced), with higher scores indicating greater imagination ability. Like Hassabis et al. (2007), participant ratings of vividness and difficulty were both rescaled from 1-5 to 0-4, and quality ratings were rescaled from 0-10 to 0-18 to calculate the experiential index.

**Measures of Theory of Mind, Autistic traits, and Emotions**

*Animations Task* (Abell et al., 2000): Participants were shown four silent animations (34-45 seconds each) of a large red triangle and a small blue triangle that are either: (i) moving randomly; (ii) moving in a goal directed fashion (e.g., fighting); or (iii) moving interactively with implied intentions (coaxing, tricking). Participants were asked “What was happening in this animation?”. Condition three is designed to elicit descriptions of the mental states of the triangles – i.e., requires mentalising. Verbal descriptions were recorded and coded. Scores ranged from 0-2 for each animation, with higher scores indicating more accurate attribution of the triangle’s mental states (e.g., desire and intentions). In our sample, scores ranged from 0-8. See Table 1 for descriptive statistics.

*Autism-spectrum Quotient* (AQ; Baron-Cohen et al., 2001): Participants were asked the extent to which they agree (definitely agree, slightly agree, slightly disagree, definitely disagree) with 50 statements relating to the self (e.g., “I am fascinated by dates”). The dependent variable is the total AQ score which can range from 0-50, with higher scores indicating a greater number of autistic traits, and scores above 26 suggesting clinically significant number of ASC traits (Woodbury-Smith et al., 2005). This has observed good test-retest reliability scores (range between *r* = .70 and *r* = .95) (Broadbent et al., 2013). In our sample, scores ranged from 4-47. See Table 1 for the descriptive statistics.

*Toronto Alexithymia Scale* (TAS-20; Bagby et al., 1994)*:* Participants were asked the extent to which they agree (ranging from 1: “strongly disagree” to 5: “strongly agree”) with 20 statements relating to understanding their own emotions (e.g., “I am often confused about what emotion I am feeling”). The dependent variable is the total TAS-20 score, which can range from 20-100, with scores of ≥61 indicating clinically significant alexithymia. This has observed good test-retest reliability (*r* = .77) (Bagby et al., 1994; Ciarrochi & Bilich, 2006). In our sample, scores ranged from 31-77. See Table 1 for the descriptive statistics.

**Hypotheses:**

1. a) Based on findings from Lind et al. (2014b) and Black et al. (2018), we predicted a general impairment in scene construction ability in autistic adults compared to neurotypical adults.

b) We predicted that several variables would correlate with scene construction ability. Specifically, we expected that participants with a higher number of autistic traits (measured on the AQ and ADOS), higher alexithymia (TAS-20 scale), and lower ToM ability (animations task) would show a reduced scene construction ability.

2. a) We predicted that sensory experiences would show a general hierarchy of mention: sight > sound > touch > taste = smell. Moreover, based on Anger et al. (2019) and the free recall nature of the scene construction task, we predicted that autistic adults would show reduced sensory experiences compared to neurotypical adults.

b) We predicted that several variables would correlate with frequency of sensory experience. Specifically, we expected that participants with higher number of autistic traits (measured on the AQ and ADOS), higher alexithymia (TAS-20 scale), lower ToM ability (animations task), and lower experiential index, would have lower frequency of sensory experience.

3. a) Based on previous empirical evidence on self-bias (Burrows et al., 2017; Grisdale et al., 2014; Henderson et al., 2009; Lombardo et al., 2007) we predicted that self-reference would be reduced in autistic adults compared to neurotypical adults.

b) We predicted that several variables would correlate with self-reference. Specifically, we expected that increased frequency of self-reference would be associated with a higher experiential index score, sensory experience and ToM (animations score), and lower alexithymia (TAS-20 score). In contrast, we did not expect a significant correlation between self-reference frequency and autistic traits (AQ and ADOS).

**Bayesian Analysis**

Bayesian analysis was also used to interpret the results (conducted using JASP 0.14.1; JASP Team, 2020), which enables a more graded interpretation than only using *p* values or effect sizes. This estimates the relative strength of the alternative hypothesis over the null hypothesis, or vice versa (e.g., Dienes, 2014; Rouder et al., 2009). For the Bayesian analyses, we adopted the default Cauchy priors as recommended by Wagenmakers *et al.* (2018). Bayes factor (BF^10^) < 1 is evidence supporting the null hypothesis (<0.33 is firm evidence), and Bayes factors > 3, >10, > 30, and >100 are firm, strong, very strong, and decisive evidence supporting the alternative hypothesis respectively.
